# What Do Students Perceive as Ethical Problems? A Comparative Study of Dutch and Indonesian Medical Students in Clinical Training

**DOI:** 10.1007/s41649-019-00101-6

**Published:** 2019-11-27

**Authors:** Amalia Muhaimin, Derk Ludolf Willems, Adi Utarini, Maartje Hoogsteyns

**Affiliations:** 1 Department of Bioethics and Humanities, Faculty of Medicine, Universitas Jenderal Soedirman, Purwokerto, Indonesia; 2 Department of General Practice, Section of Medical Ethics, Amsterdam University Medical Centre, University of Amsterdam, Amsterdam, The Netherlands; 3 Department of Health Policy and Management, Faculty of Medicine, Public Health and Nursing, Universitas Gadjah Mada, Yogyakarta, Indonesia

**Keywords:** Ethics education, Medical ethics, Student perception, Clinical training

## Abstract

Previous studies show that medical students in clinical training face ethical problems that are not often discussed in the literature. In order to make teaching timely and relevant for them, it is important to understand what medical students perceive as ethical problems, as various factors may influence their perception, including cultural differences and working environment. The purpose of this qualitative study was to explore students’ perceptions of what an ethical problem is, during their clinical training in the hospital, and compare the results from two different countries. We observed a total of eighteen ethics group discussions and interviewed fifteen medical students at two medical schools, in Indonesia and the Netherlands. Data were interpreted and analyzed using content analysis. We found that students in both settings encounter problems which are closer to their daily work and responsibilities as medical students and perceive these problems as ethical problems. Indonesian students perceived substandard care and inequity in healthcare as ethical problems, while Dutch students perceived that cases which are not matters of life and death are less worthy to discuss. Our study suggests that there might be a gap between ethical problems that are discussed in class with teachers, and problems that students actually encounter in practice**.** Teachers should be aware of the everyday situations in clinical training which may be perceived by students as ethically problematic and should acknowledge and discuss these ethical problems with students as part of the learning processes in ethics education.

## Introduction

Twenty years ago, a third-year Canadian medical student addressed the importance of acknowledging student-specific ethical dilemmas in the curricula, and stated that most contemporary ethics curricula in medical schools have failed to address these specific issues (St. Onge 1997). More recent studies showed that general practitioners have different perceptions on how terms like *ethical problem* and *ethical dilemma* apply in everyday practice (Braunack-Mayer 2001), while a study in Germany shows that teachers in medical schools were unable to identify ethical issues encountered by students in medical training, although they were familiar with ethical issues in healthcare (Chiapponi et al. 2016). Clearly, there is a persisting question on what medical students perceive as ethical problems and what they need from ethics education (Eckles et al. 2005). The concept of an ethical problem has actually been supported by a large body of literature in the field of bioethics and medical ethics. However, the concept and definition have developed since the old days of medical ethics and Hippocratic Oath, to the modern days of Bioethics (Kuhse and Singer 2009), while other literatures introduce the more narrowed definition of ethical problems as ethical dilemmas, implying that problems of conflicting moral principles and choices are essential (Beauchamp and Childress 2008; Jonsen et al. 2006; Lo 2013). In our experience as ethics teachers, we noticed that students often have difficulties to pinpoint ethical problems and they come up with different, often culturally and socially shaped, concepts of what constitutes ethical problems. Different health and education systems may add to that. Hence, medical students working in different settings may have different perceptions of what an ethical problem is. The varying “conceptual starting points” of students are at the same time crucial for the effectiveness of teaching and have not been explored. For this reason, we were interested in exploring what medical students perceive as ethical problems and compare students’ perceptions from two different countries.

In the Netherlands, the Dutch Federation of University Medical Centres (NFU) published a framework to define the learning outcomes of Master programs in Medicine (Laan et al. 2009) elaborated from the CanMEDS 2015 Framework (Frank et al. 2015). In Indonesia, learning outcomes for graduates of medicine are formulated in the Standard of Competencies for Indonesian Physicians or the so-called *SKDI*, where “ethics and professionalism” is the cornerstone for the overall competence of medical doctors (Konsil Kedokteran Indonesia 2012). Both frameworks suggest that physicians should be able to recognize ethical problems and make ethical decisions in healthcare practice. Despite similarities in framework, learning goals, and teaching strategies, previous discussions among ethics teachers and clinical teachers in the national and international level indicate differences in the cases or problems they bring up. In Indonesia, medical ethics is very much nuanced by issues of ethical behavior, professionalism, and medicolegal, while ethical discourse in the international level more often discuss complex ethical dilemmas involving advance medical technologies and global health problems. Moreover, bioethics is relatively new in Indonesia, and therefore, not many teachers in medical schools are trained in ethics. These differences suggest that there might be different perceptions among students on what an ethical problem is. This is important for teachers to understand, to be able to identify students’ learning needs, especially during their clinical training phase (clerkship) in the hospital. If we can understand what students perceive as ethical problems, then we will be able to identify relevant topics and develop the appropriate learning goals and strategies for ethics teaching. For this purpose, we conducted a qualitative study at two medical schools: in Indonesia and The Netherlands.

## Methods

### Organization

We have chosen two medical schools in this study due to similarities in the learning strategies used for ethics teaching in the clerkship phase. Both schools regularly conduct ethics discussions for clerkship students (“clerkship” denoting the clinical training phase) as part of their curriculum. The two schools have been collaborating to improve ethics teaching and facilitators from both schools have had similar training to facilitate ethics discussions and conduct ethical deliberations. Hence, despite slight differences in organization, both have important similarities in the teaching methods used, which is crucial for our study. First, ethics discussions in both settings are organized by a special unit or department dedicated for medical ethics. Second, they are conducted in small groups, facilitated by ethics teachers (not by clinical teachers), and conducted outside of their clinical learning environment. Third, students are free to choose their own ethical cases or problems, which they have encountered during their clinical training. Students are not given a certain case nor presented with any scenarios from their teachers. The fact that students choose and discuss their own cases shows the concepts of ethical problems they use.

For this study, we have kept the schools anonymized to avoid any consequences for research subjects as well as healthcare providers. Subjects are clerkship students, called “coassistant” in The Netherlands, or “koasisten” in Indonesia, working in a tertiary (referral) hospital. In the Netherlands setting, ethics discussions are conducted in three different sessions throughout the clerkship program, facilitated by ethics teaching staffs from the Section of Medical Ethics, Department of General Practice. It starts with a brief introductory session, followed by a case discussion a few weeks later, and a second round of case discussion in the third clerkship year. In all three sessions, students are asked to share their own ethical cases. In the Indonesian setting, students have one session of ethics discussion during their round at the Department of Forensics and Medicolegal, conducted in two consecutive meetings, facilitated by teaching staffs from the Department of Bioethics and Humanities. During the first meeting, students discuss briefly their own cases which they have submitted beforehand, and then together choose the two most interesting cases to discuss in-depth during the second meeting a week later.

### Data Collection

Data were collected from March 2016 to August 2017. We conducted 18 participant observations, ten in the Netherlands and eight in Indonesia (Tables 1 and 2), and 15 in-depth interviews with seven Dutch students (C1–C7) and eight Indonesian students (K1–K8). For this study, classes in the Netherlands were carried out in English with students’ agreement, while in Indonesia classes were carried out in Indonesian Language (*Bahasa Indonesia*)*.* Dutch students were always free to speak Dutch if they had any difficulties, and facilitators or other students were always helpful to translate the words or main ideas into English.

**Table 1:** Participant observations in the Netherlands

Group	Participants	Gender		Cases discussed
		Female	Male	
A. First session—Introduction class (30 min)				
I	14	8	6	2
II	14	7	7	2
III	15	7	8	2
B. Second session—Case discussion (60 min)				
IV	9	6	3	5
V	8	3	5	2
VI	12	5	7	12
C. Third session—Case discussion (180 min)				
VII	9	4	5	9
VIII	9	4	5	9
IX	12	9	3	3
X	10	9	1	10
Total	112	62	50	56

**Table 2:** Participant observations in Indonesia

Group*	Participants	Gender		Cases discussed
		Female	Male	
Ia	8	4	4	8
IIb	13	4	9	3
IIIa	8	5	3	4
IIIb				
IVa	9	7	2	9
IVb				
Va	12	7	5	12
Vb				
Total	50	27	23	36

*Observation was conducted in both sessions or either one (a = first session; b = second session)

Students were informed about the study and were asked permission to have the discussion audio recorded and field notes taken. We explained that any data collected will be kept anonymous and unidentifiable to ensure both students’ and facilitators’ privacy and confidentiality. After each class, students were contacted through e-mail or text message and asked for an in-depth interview. A written consent, each in English and Bahasa Indonesia, was obtained at the time of interview.

The semi-structured interviews were approximately 50–100 min in length. Students were given the chance to recognize and define the ethical problems themselves. The interviews began with the question: “Could you tell me about an ethical problem you have experienced during your clerkship?” Questions were then formulated according to the interviewee’s narratives and responses.

### Data Analysis

All interviews, observations, and coding were done by AM, and analyzed using content analysis (Silverman 2006). Coding and analysis of the first three observations and interviews were checked separately by DW and AU and discussed until consensus was reached. We interpreted and analyzed students’ experiences to understand what they perceived as ethical problems. After thirteen observations and interviews, respectively, no new data were found, nor new codes were needed. In order to ensure data saturation, we conducted two more observations and two more interviews to ensure no new categories nor themes were obtained. This practice is important to ensure data saturation was reached (Fusch and Ness 2015)**.** In this paper, we first grouped the cases based on existing literature to give a general description (Dickenson et al. 2010; Jonsen et al. 2006; Kushner and Thomasma 2001)**.** We then analyzed how students discussed the problems, what makes the problem ethical for them, and categorized their perceptions (Table 5). Some details of the cases have been modified to protect privacy and confidentiality of patients, healthcare workers, and students. Interpretations were sent to participants to ensure their perspectives are correctly represented (Tong et al. 2007; O'Brien et al. 2014)**.** Twelve out of fifteen students who were interviewed sent back their comments and none of them disagreed with the interpretations.

## Results

The results of this study are presented in three sections. The first section is intended to give a general description of what kind of cases students shared in each setting (Tables 3 and 4). The second section shows students’ perceptions of uncertainties regarding the concept or nature of what an ethical problem is. Finally, the third section presents five categories on what students perceive as ethical problems (Table 5) or what the nature of ethical problems are. In other words, what makes the problem an ethical concern from their point of view.

**Table 3:** Cases submitted by the Dutch students

No**.**	Topics	Subtopics	Case description
1.	Privacy	Privacy	- Installing secret camera at home
2.	Forced feeding	Forced feeding	- Patients with anorexia
3.	Refusal of treatment	Refusal of treatment	- Patients refusing cesarean section - Schizophrenic patient with terminal renal disease - Patient with malignancy refuses surgery - Patient’s spouse refuses adequate pain management - Parents refuse cochlear implant for their child - Psychiatric patient refuses birth control
4.	End of life	Withholding/withdrawing treatment	- Suicidal patient in critical condition - Newborn twin with severe illness
		Request for euthanasia/ physician-assisted suicide	- Patient with obsessive compulsive disorder - Family’s request of patient with Alzheimer - Patients with depression
5.	Professional secrecy	Confidentiality	- Prisoner with guard in the examination room - Asylum seeker with shared psychotic disorder - Male patient discovered as female - Child with bruises, suspect of child abuse - Baron von Munchausen syndrome
		Obtaining data without patient’s consent	- HIV test request from fireman rescuer - Patients with sexual transmitted disease
6.	Procreational decisions	Sperm donation	- Patient with infertility - Single parents/unmarried/homosexuals
		Sex selection	- Facilitating ultrasonography for later sex selection
7.	Student’s role and responsibility	Student’s duties and responsibilities	- Informing serious diagnosis to patients - Drunk teenager with leukemia - Patient wanting to sue doctors in-charge - Copying patient’s medical record - Student referring patient to hospital by car
		Questioning decisions from seniors	- Doctor sending ambulance instead of visiting patient - Neglecting patient’s request for pain management - Surgeon relying on examination from student - Korsakoff syndrome with stroke symptoms - Late referral, newborn with hiperbilirubinemia
		Being professional	- Refuse to take pictures with the patients - Communicate with the patient’s family

**Table 4:** Cases submitted by the Indonesian students

No.	Topics	Subtopic	Case description
1.	Privacy	Privacy	- No privacy for patients at the lowest class ward
2.	Professional secrecy	Confidentiality	- Patient with HIV: disclosing information to other healthcare workers
3.	Refusal of treatment	Refusal of treatment	- Retentio placentae: patient’s refusal for treatment - Intracranial hemorrhage: family’s refusal for surgery - Peritonitis and laparotomy: family refused surgery - Child with epidural hematoma: family refused surgery - Diabetic coma: family’s refusal for resuscitation - Lower limb fracture: patient and family’s preference for alternative treatment
4.	Patients without the capacity to consent	Disclosing information to family Incidental/unexpected findings	- The inmate with epidural hematoma: whose consent? - The 17-year-old girl with abdominal pain: should we tell the parents? - Hernia inguinalis: family’s consent and death in the operating room - Atonia uteri: hysterectomy using husband’s consent
5.	Lack of resources	Healthcare insurance Doctors’ working hours	- Patients who cannot afford to pay - Problems dealing with the healthcare system and insurance company - Limited medication/treatment for patients covered by the national healthcare insurance - Doctor’s workload and working hours
6.	Quality of care	Unprofessional behavior	- Being disrespectful to patients - Congenital disorder: doctor blaming the parents - Blaming healthcare workers in front of the patient - Severe head injury: late arrival of the consultant
		Substandard care	- Cesarean section: evaluating a death case - Steven Johnson Syndrome: neglected patient - Patient’s death and family’s disappointment
7.	Student’s role and responsibility	Student’s duties and responsibilities	- Delivering bad news to patients and families - Complex bureaucracy and bending the rules - Dealing with conflicting orders
		Questioning decisions from seniors	- Taking family’s consent for non-treatment - Admitting patients to the ward - Filling in medical records
		Training hierarchy and teamwork	- Being inferior and question of authority - Taking the blame from doctors and nurses Keeping quiet and covering up mistakes

**Table 5:** Students’ perceptions of ethical problems

Coding (Dutch students)	Categories	Coding (Indonesian students)
Dilemmatic situation Conflicting opinions Conflicting choices Conflicting values Right or wrong Gray area	Conflicting choices	Dilemmatic Conflicting opinions
Rights Duties Responsibilities Professionalism Standards and regulations	Duties and responsibilities	Rules/regulation Standard procedure Question of authority Being professional Teamwork Being inferior Problems to communicate
Frustration Helplessness Difficult situation Something not right Emotionally difficult	Emotionally disturbing situations	Guilty Upset Resentful Speechless Disappointed Helpless Angry
Neglecting patients Unprofessional behavior	Problems of justice and quality of care	Inability to pay Lack of resources Health care system Unprofessional behavior Treating patients equally Poor quality of care Neglected patients Medical errors
Patient was not dying Not a matter of life and death	Life-threatening cases	–

### Description of Cases

Tables 3 and 4 give an overview of the cases. In the Indonesian setting, many cases were related to lack of resources and quality of care, while cases related to *forced feeding*, *end of life*, *and procreational decisions* were not present. Although most cases involved patient care, the ethical problems brought up by the students did not necessarily involve the patient, such as cases related to students’ role and responsibilities, where the conflicts were mainly between students and their supervisor or other healthcare workers.

### Is it an Ethical Problem?

Students in both settings were sometimes uncertain whether their cases were regarded as ethical problems. A Dutch student described that she did not know at first what an ethical problem is, when she was asked to submit a case. She asked her fellow student about this, but her friend was also not sure about it, because her case was not considered a real ethical problem by the facilitator when it was discussed in class.

…and then when he (the facilitator) came to her case, they discussed it... He said, you know, when you check the criteria, this is not really an ethical problem. So he explained that, so that you know. That is nice for her because otherwise she doesn't know... (C4, female, 3rd year clerkship)

One of the students in Indonesia also expressed his doubts, when he was asked if he had any ethical problems to share with during the interview:

Hmm… ethical problem… yes, I do have some… the first has to do with the training system, being a coassistant, so it’s not between a doctor and a patient… is that okay? I also have one about scheduling night shifts… but I’m not sure if it fits (as an ethical problem)… (K6, male, final year clerkship)

The student wanted to make sure that the case he was going to share was regarded as an ethical problem by the interviewer. After being informed that he was free to share any cases he perceived as an ethical problem, he then shared a case about his conflict with a nurse, who is known for bullying students in the operation room. Once, he had had the courage to kindly speak up to the nurse about this, which surprised the nurse. His ethical issue was that his action, while good in itself, might bring harm to himself or his fellow students.

Both students had doubts on whether or not their cases would be considered as an ethical problem by their supervisor (in the Dutch case) or by the interviewer (Indonesian case). During the case discussions, facilitators in both settings did not always point out explicitly if the problems students brought up were indeed an ethical problem or not. They would more often help students formulate the ethical problem, especially in the Indonesian setting, where students often have not yet formulated the ethical problem in their case reports.

### Students’ Perceptions

Five categories emerged from both settings, three of which were more prevalent in either setting; i.e., “problems of justice and quality of care” was more prevalent among the Indonesian students, while “conflicting choices” and “life-threatening cases” were more prevalent in the Dutch setting.

#### Conflicting Choices

Many Dutch students perceived ethical problems as conflicting choices. During an interview, a student shared a case which he also submitted for the class discussion, about a 60-year-old woman who was a healthcare worker and had acute anemia. The medical team wanted to perform an endoscopy to find the source of bleeding, but the patient refused and instead wanted to have more blood transfusion. When asked what was most interesting and ethical about the case, he explained his perception of an ethical dilemma with conflicting choices:

I think an ethical dilemma is something that is going the other way than you want … So I want to go to route A and the patient wants to go to route B… and it’s not the same route… because as a doctor, I think route A is the best, but the patient thinks, ‘no… route A is the worse and I want to go to route B’… (C5, male, 1st year clerkship)

Another Dutch student described his case as a “gray area” between right and wrong, which cannot easily be determined using common norms or standards. The student spoke about his parents who wanted to install a video camera secretly at his grandparent’s home to monitor their condition, without informing the grandparents nor their assistant. The idea was based on good intentions and may seem right, but it can be considered as a wrongful act because it violates the privacy of others.

I think it is (difficult) because there is an ethical question… it’s a gray area, between right or wrong. (Introduction class, male, 1st year clerkship)

An Indonesian student, K5, also shared his experience during a night shift in the ER. The surgical resident on duty that night told K5 to refuse a patient with severe head injury who was about to be referred to the hospital because he had to operate another patient. However, the ER doctor insisted that they receive the patient because they were a referral hospital in the region.

(And the resident said) ‘Please tell the ER doctor that we are full here’… So I was really confused… what should I say to him… if I said yes, but the patient still came... there was only one resident here, and he will have to do it… but if I say no (disagree with the resident), I might get into trouble… And that was really the most dilemmatic moment of my clerkship… (K5, male, final year clerkship)

The student decided to tell the ER doctor as he was told to and was thankful that the ER doctor insisted in receiving the patient. Even if they could not operate the patient immediately, they could take care of the patient better than the previous hospital which had less resources.

#### Duties and Responsibilities

Students in both settings brought up ethical problems related to the student’s role and professionalism. A Dutch student shared his case about a young mother in her early pregnancy who had severe headaches and fever. During the course of the illness, she suffered from severe pain in the legs. The doctors suspected sarcoidosis but could not perform radiological tests nor give any therapy because they were teratogenic. As much as he wanted to help the patient and her husband, he found himself helpless and accidentally said something which he had deeply regretted.

... So in the end I said, well, with a lot of frustration out of myself, I said to him (patient’s husband): ‘Well, I wish we could do so much more... I wish we could just get the diagnosis or something’, and then I just basically walked away... (C3, male, 1st year clerkship)

He had questioned his role, his duty and responsibility, as a medical student and a future doctor. He discussed the importance of communication and professionalism in medical training and how it should be emphasized in ethics education. In another interview, an Indonesian student shared her concerns about students’ task to get the patient’s or family’s signature on the informed consent form. She thought that such important tasks should be done by a physician or nurse who has a legal capacity. In many cases, the patient and family have not been given sufficient, if not any, information about the procedure.

Well… it (the informed consent process) is often simplified… and the students are the ones who must get the signature, and even give the information… whereas I think this is an important thing, with legal implications… so an official hospital staff should be the one who can do it properly, to inform everything about the procedure, including the risks… (K2, female, 2nd year clerkship)

Indonesian students shared similar cases, where they questioned orders from seniors, and question if students are allowed or competent to do certain procedures. Such as K7 who shared a case about a patient with generalized peritonitis, whose condition was deteriorating with severe pain. The resident on duty that night told K7 to inform the family that the patient was in bad condition and that there was nothing else they can do. She questioned whether such tasks, such as delivering bad news and taking consent for non-treatment, should be carried out by students.

#### Emotionally Disturbing Situations

Students from both settings shared cases particularly because they were emotionally disturbing. This category might be seen as somewhat overlapping with the previous (conflicting choices); however, we consider this as a specific category because not all students felt the problem that they brought up was emotionally disturbing for them. A Dutch student shared a case, which she also submitted for the group discussion, about a young woman with extensive psychiatric history who lived in an institution. The health personnel suggested the patient to use birth control. However, she refused and expressed her desire to have children. The student stated that her reason for choosing the case was because it was emotional, and it was the only case which really had a strong impression on her.

It was more emotional… because it’s closer (to me)… For me it wasn’t about the birth control… It was more like… emotional, the whole patient and everything, and it wasn’t confined to one (ethical) question… because you’re intensely working with someone for weeks with such problems, you know… (C7, female, 3rd year clerkship)

In another interview, an Indonesian student, K4, shared her experience in the ER, when a 7-year-old boy was admitted with a persistent headache due to a head injury the day before. The child was diagnosed with epidural hematoma and planned for surgery, but the parents said they needed some time to think before giving consent. The nurse, student, and resident tried to convince the parents that the child needed surgery urgently. But the parents insisted on bringing the child home and wanted to try an alternative (spiritual) medicine called “ruqyah.”

At that time, I felt resentful (‘sebal’) because we had warned them over and over that there is no medication for this, other than surgery; to remove the bleeding and repair the damaged blood vessel. But they still did not give consent… so I was very upset, and… well… everyone has the right to determine or decide what they want... (K4, female, final year)

K4 said that she felt helpless and regretted not finding out why the parents refused, whether it was due to their beliefs or economic reasons. She felt guilty and said that they should have done more to protect the child, although she doubted that they could perform surgery without the parents’ consent.

#### Problems of Justice and Quality of Care

Many Indonesian students perceived inequity, lack of resources, and poor quality of care as ethical problems, while this category emerged far less from the Dutch students. One of the cases shared during the group discussion was about a young woman who was referred to the hospital by a midwife because of a *retentio placentae.* The midwife in the hospital tried to remove the remaining placenta. However, the patient said that she could not stand the pain and refused to continue the procedure. The patient’s husband said that it was up to his wife, and after discussing with the patient’s father, they decided to bring the patient home because she wanted to stay at home to take care of her other children because her husband had to work. Moreover, they did not have any health insurance. Another student also shared her concerns about the outpatient clinics in the hospital, where there are insufficient doctors for the patients, who mainly come from the lower class. In some outpatient clinics, there are more than 100 patients per day with only 2–3 min per encounter. The service is often not worth the long travel and waiting hours. Some patients complain and question the doctor’s service to the students, and students become very uncomfortable and have no idea how to respond.

Another case was about a young man admitted to the ER after a traffic accident. The patient was alert but suffered from progressive epigastric pain and was suspected to suffer from internal bleeding. He was put on waiting list for a laparotomy because there were other emergency operations and only one resident on duty. Unfortunately, the patient’s condition deteriorated, and he died during the operation. The doctor then reminded everyone in the OR, especially the two students, not to disclose the incident to anyone. The students were told to bring the patient to the ICU, where he was to be declared dead. The students first agreed to keep quiet, but they eventually disclosed the case to their clerkship group to remind them that they need to evaluate patients more carefully to avoid similar cases. Through the discussion, we found that the case was very complicated, not only involving fraud and neglect but also complex bureaucracy and high workload of residents and students.

#### Life-Threatening Situations

During an interview, a Dutch student shared her experience working with her supervisor, who asked her to bring a patient with chest pain to the hospital by car. She realized later that it was not the right thing to do, and that they should have called the ambulance. When asked why she did not tell her supervisor afterwards about her thoughts, she said that it was not a matter of life and death, and luckily everything went fine.

Well umm... that’s a good question… at that time I was like, okay, she’s not going to die, but it doesn’t feel good... and she had to have more examinations, so that’s why we decided to send her to the hospital... But... afterwards, I was like hmm... this is not the best idea ever... no... I shouldn’t do this... (C2, female, 3rd year co-assistant)

Another student, C1, also brought up a case that she perceived as an ethical problem but did not dare to speak up about, because it was not really a matter of life and death.

I don’t know... I just thought it was not right or something... but it’s not really a matter of life and death or something so that makes it... (thinking…) like if it was a matter of life and death, then I would of course say it to someone... but this was just really subtle*...* (C1, female, 3rd year clerkship)

Both students did not directly say that an ethical problem should be a matter of life and death. However, they thought that if they are not, they are not worth discussing with teachers and supervisors. In other words, if a problem is not life-threatening, then it is not a “real” or “serious’ ethical problem.

## Discussion

### General Description of Cases

In this study, we found differences, as well as similarities, between the two countries with regard to the cases students shared (“Description of Cases”), although the differences do not indicate that those cases occur only in either setting. The topic of professional secrecy, for instance, was brought up more often by Dutch students. Although in Indonesia professional secrecy is regulated by the law (Indonesia 2009, 2012), the current situation suggests that privacy and confidentiality have not yet become a major concern for patients. This is perhaps due to the collective culture in Indonesia, and this might explain why fewer ethical problems were related to the issue of professional secrecy in the Indonesian setting. Topics of euthanasia and procreational decisions were also brought up more frequently by Dutch students, and hardly by Indonesians. This is not surprising given that euthanasia is illegal in Indonesia (Pradjonggo 2016), while many procreation-related technologies are highly controversial (Indonesia 2014), and therefore leave no space for such cases to occur in the hospital. Other topics, such as “lack of resources” and “quality of care”, were more often brought up by Indonesian students. Students in the Dutch setting did not bring up any cases related to problems of healthcare access and limited resources. Although a few Dutch students questioned quality of care and decisions made by their seniors, their major concerns or questions were about their own role and responsibility as a medical student in that given situation, whereas Indonesian students often shared cases related to lack of resources, substandard care, and unprofessional behavior as a reflection and concern of witnessing similar cases in the hospital in their daily work.

Similarities were also found, where two topics were often brought up in both settings, namely, “refusal of treatment” and “student’s role and responsibility.” However, there were contextual differences among the cases between the two countries. In the Dutch cases, patients or families refused treatment due to reasons of best interest for themselves or for their loved ones, while cases of refusal in the Indonesian setting were often caused by financial problems due to the patient’s (or patient’s family’s) inability to cover healthcare costs, including problems with the healthcare insurance. These patients were autonomous, have the capacity for self-determination, but could not act or decide on the grounds of their best interest due to financial constraints. These reasons were not present in the cases from the Dutch setting. The topic of student’s role and responsibility in the Indonesian setting also differed slightly from the Dutch, where it was more nuanced with issues of authority, hierarchy, and complex bureaucracy, compared to the Dutch setting. Hence, cases from Dutch students involved fewer conflicts between students, supervisors, and other healthcare workers in the hospital. This general description of the cases students shared is intended to show the wide range of clinical cases students perceive as having an ethical problem, as often described in existing literature (Kuhse et al. 2015; Lo 2013). However, it does not really show what makes the problem an ethical problem from the students’ point of view. Our study shows that students were sometimes uncertain on whether or not the problems they shared could be considered as ethical problems (“Is it an Ethical Problem?”). Although supported by a large body of literature, what is to be regarded as an ethical problem is sometimes unclear for students or perceived differently.

### Perception of Ethical Problems

Similarities and differences from the five categories were also found between the two countries (Table 5). The majority of Dutch students perceived ethical problems as ethical dilemmas with conflicting choices, far more than Indonesian students, and some perceived that ethical problems which are not life-threatening are less worthy of discussion with teachers and supervisors. This is possibly due to a number of reasons. First of all, students came from two different social and cultural backgrounds, and worked in two different healthcare systems. Second, there was a difference in the learning process. Dutch students were given a set of criteria for their case reports by their facilitators, one of which lead to an ethical dilemma, while Indonesian students had more freedom to choose any case which they felt problematic. Despite the given criteria, from the interviews, we found that Dutch students did not always perceive ethical problems as dilemmas. Previous studies have discussed how to trigger students to bring up ethical problems (Donaldson et al. 2010; Kaldjian et al. 2012). The advantage of having more distinctive criteria, as it is in the Dutch setting, is that the discussion can be more focused on achieving certain learning goals, for instance, to resolve ethical dilemmas. However, with more open criteria, as it is in the Indonesian setting, students do not feel obliged to select a case which best fits the criteria, while the goal is more focused on broadening perspectives.

Many Indonesian students perceived problems of justice and poor quality of care as ethical problems, while this category was mentioned far less by Dutch students. Concerns of quality care in the Dutch setting were more often due to unprofessional behavior of healthcare workers rather than lack of resources, although concerns of unprofessional behavior was far more prevalent in the Indonesian setting. We believe that substantial social and cultural differences between the two countries, including the healthcare system, play a major role in constructing students’ perception, as they also influence the kind of cases students encounter. However, complicated ethical problems regarding healthcare systems and policy which are predominant in developing countries remain a difficult topic and are rarely discussed in the literature (Iserson 2018; Iserson et al. 2012). Another difficult topic which emerged quite frequently from the Indonesian setting was about unprofessional behavior from healthcare workers leading to poor quality of care (Chaudhury et al. 2006). Vidal stated that unprofessional attitudes and behavior in healthcare institutions are rarely reported due to fear of retaliation and lack of anonymity of reporting mechanisms (de Oliveira Vidal et al. 2015; Caldicott and Faber-Langendoen 2005). Furthermore, the organizational culture in Indonesia shows a large power distance within the hierarchy (Irawanto 2009), with students being inferior, often causing uncertainties and barriers in communication (Muhaimin et al. 2012). We suggest that such problems should be addressed and discussed by teachers and students, as it may cause declining empathy and ethical erosion of medical students if not handled properly (de Oliveira Vidal et al. 2015).

Despite the differences, students in both settings perceived ethical problems as problems related to duties and responsibilities, in particular related to their role as clerks. Similar to our study, ethical problems among third-year medical students in the United States were also related to daily problems concerning professional duties and specific issues (Caldicott and Faber-Langendoen 2005; Kaldjian et al. 2012), while Kushner and Thomasma’s *Ward Ethics* (Kushner and Thomasma 2001) also discusses the “hidden” problems related to students’ duties and responsibilities, describing their discomforts and distress in everyday clinical training. Sturman’s study in Australia suggests that teachers should explore more common ethical issues which are relevant to students (Sturman et al. 2014). Students in our study also perceived ethical problems as emotionally disturbing situations. They feel closer and more emotional when dealing with cases where they have more responsibility. Unfortunately, mainstream bioethics literature and models of ethical deliberation provide relatively little room for emotions. A study by Guillemin and Gillam discusses the importance of emotions and ethical reflection in ethics education (Guillemin and Gillam 2015; Guillemin et al. 2009), suggesting that emotions are important for moral sensitivity, to be able to recognize situations which are ethically problematic. However, addressing emotional responses can be difficult for medical students as they may feel embarrassed or fear that they would be judged negatively by their seniors.

Students’ responses in our study suggest that, for medical students, the ethical domain may be broader than ethical dilemmas and conflicting choices, and includes definitions focused on responsibilities, emotions, notions of justice and quality of care. Despite similarities in the educational framework and source of teaching materials, we found differences in perceptions among clerkship students between two different countries. These differences suggest that social and cultural context play an important role in students’ definitions of ethical problems. Nevertheless, students in both settings also shared similar problems and concerns with regard to their learning environment, especially related to their role and responsibilities as medical students.

## Conclusion

Our study suggests that there may be a gap between ethical problems that are discussed in standard teaching classes and problems encountered by medical students in the real setting. We believe it is important for students to be able to share those problems, and for teachers to be aware and open to how students themselves define or recognize ethical situations to facilitate shared learning. In doing so, they can learn from students as well. Defining ethical problems rather narrowly as ethical dilemmas or conflicting choices might only capture a small range of medical students’ views about the nature of ethical problems. Most importantly, we need to consider that there may be different approaches to understand the nature and process of ethics discussion and moral deliberation for the purpose of ethics teaching, especially in different settings. Hence, learning strategies should be adapted to accommodate everyday situations in clinical practice that are perceived by students as ethically problematic.

## Limitations

In the Dutch setting, all classes observed, and all interviews were conducted in English, which is not the native language of the researcher nor the participants. Hence, slight misinterpretations might have occurred, although students rarely spoke Dutch during the classes (despite the opportunity given) and spoke English fluently. This study was conducted in two academic hospitals, and therefore, the results might or might not be similar elsewhere.
